# Supplementary figures and images for: Increased soluble amyloid-beta causes early aberrant brain network hypersynchronisation in a mature-onset mouse model of amyloidosis
Source: Acta Neuropathol Commun. 2019 Nov 14;7:180. doi: 10.1186/s40478-019-0810-7 (PMC6857138; doi:10.1186/s40478-019-0810-7)

Default mode-like network

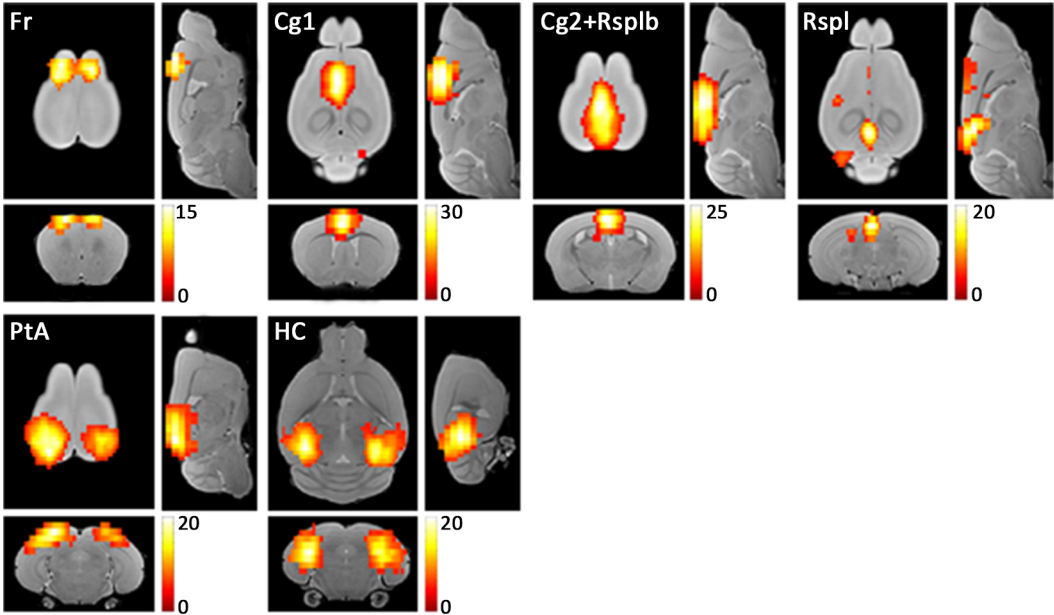

Sensory network

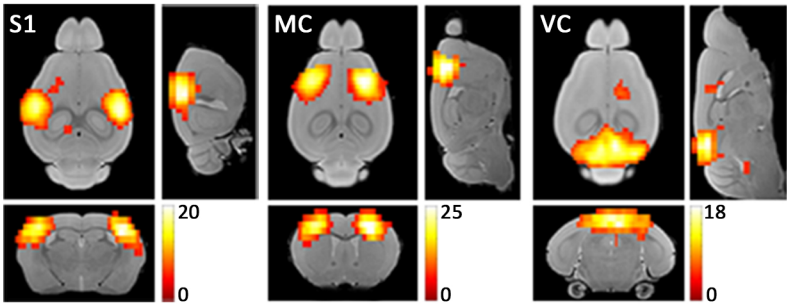

Subcortical network

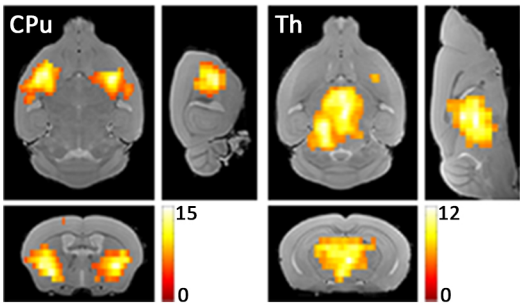

Supplement: Supplementary file 1 — Additional file 1: Figure S1. Overview of the ICA components of interest. Statistical maps are overlaid on an anatomical 3D template. The colour scale indicates t-values, with yellow representing a stronger correlation. An FWE correction of p < 0.05 and a minimum cluster size of 10 voxels was applied. Abbreviations see Table 2. [file 40478_2019_810_MOESM1_ESM.pdf]

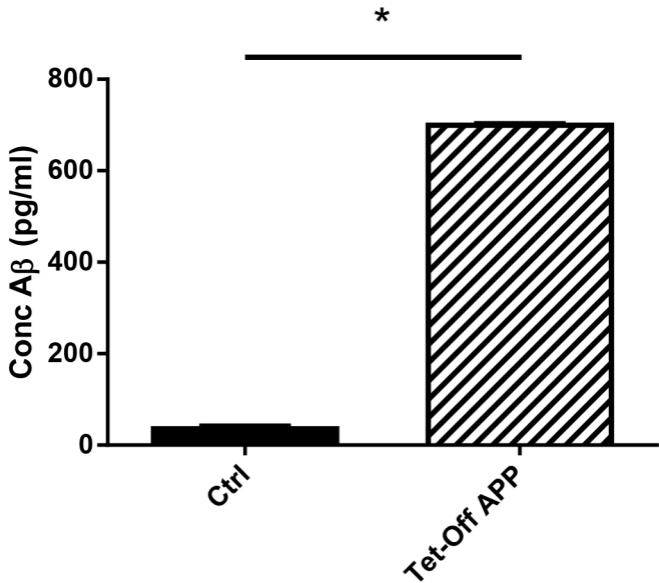

Supplement: Supplementary file 3 — Additional file 3: Figure S3. Total sAβ levels in the Tet-Off APP model. The TG animals showed a significant increase in total sAβ levels which were about 20-fold higher compared to the controls (NTG = 3, NCTRL = 6). All values are presented as mean ± SEM; striped bar corresponds to TG group and black bar to the Ctrl group. The significant Group effect is indicated with stars. *p < 0.05. [file 40478_2019_810_MOESM3_ESM.pdf]
