# Supplementary material for: Increased soluble amyloid-beta causes early aberrant brain network hypersynchronisation in a mature-onset mouse model of amyloidosis
Source: Acta Neuropathol Commun. 2019 Nov 14;7:180. doi: 10.1186/s40478-019-0810-7 (PMC6857138; doi:10.1186/s40478-019-0810-7)
Supplement: Supplementary file 2 — Additional file 2: Figure S2.Time evolution of FC in regions of the DMN network. Each graph shows the FC over time of a specific region. Results are shown as mean ± SEM. Significant [Genotype*Time] interaction and Time effect are indicated with stars and dotted line, respectively. *p < 0.05; **p < 0.01; ***p < 0.001; ****p < 0.0001. [file 40478_2019_810_MOESM2_ESM.pdf]

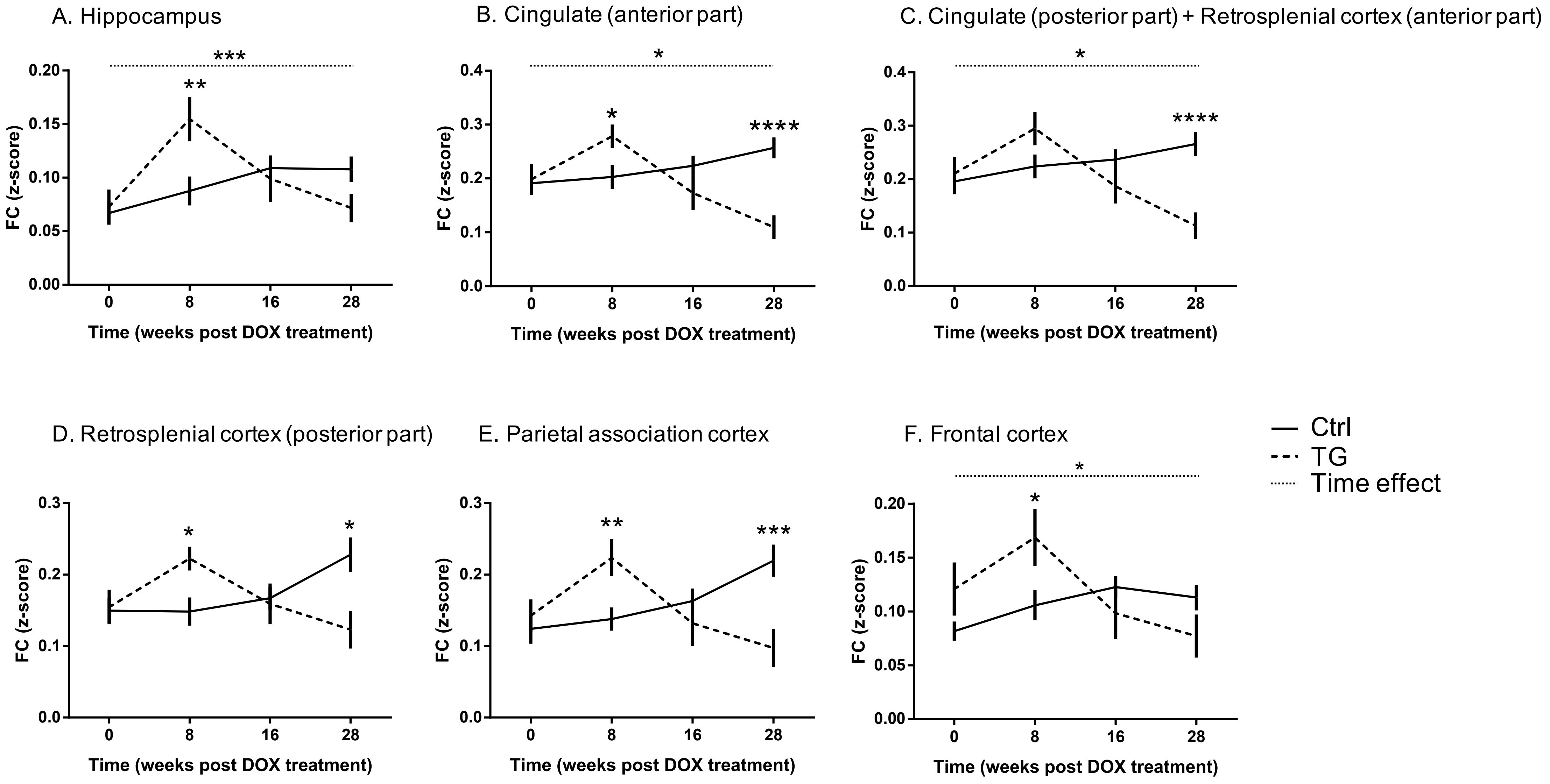

A. Hippocampus
B. Cingulate (anterior part)
C. Cingulate (posterior part) + Retrosplenial cortex (anterior part)
D. Retrosplenial cortex (posterior part)
E. Parietal association cortex
F. Frontal cortex
FC (z-score)
Time (weeks post DOX treatment)
0
8
16
28
Ctrl
TG
Time effect
